# Function of Nodulation-Associated GmNARK Kinase in Soybean Alkali Tolerance

**DOI:** 10.3390/ijms26010325

**Published:** 2025-01-02

**Authors:** Huiying Ma, Xin Liu, Rui Zhang, Minglong Li, Qiang Li, Xiaodong Ding, Jialei Xiao

**Affiliations:** Key Laboratory of Agricultural Biological Functional Genes, Northeast Agricultural University, Harbin 150030, China

**Keywords:** GmNARK kinase, alkali stress, organic acids, ROS

## Abstract

Soybean (*Glycine max*) is a vital crop that is rich in high-quality protein and edible oil for human nutrition and agriculture. Saline–alkali stress, a severe environmental challenge, significantly limits soybean productivity. In this study, we found that the nodule receptor kinase GmNARK enhances soybean tolerance to alkali stress besides nodulation. *GmNARK* could be induced by alkali stress in soybean roots. Ectopic overexpression of the *GmNARK* gene in Arabidopsis could significantly improve plant tolerance to alkaline stress. Moreover, overexpression or silencing of the *GmNARK* gene in soybean hairy roots also enhanced composite soybean plant tolerance to alkaline stress on plates and in soils. Additionally, overexpression of the *GmNARK* gene upregulated expression levels of the genes that are involved in the reactive oxygen species (ROS) signaling pathways. These findings provide a critical theoretical basis for further elucidating the role of GmNARK kinase in salt–alkali resistance and lay a foundation for improving soybean productivity under salt–alkali stress.

## 1. Introduction

The aggravation of soil salinization in arable land greatly limits the growth and development of crops, which has serious consequences for the sustainable development of agriculture [1]. Saline–alkali soils are typically characterized by high salinity and elevated pH (pH > 8.0) [2], which induce osmotic stress and ion toxicity in plants [3]. In response to salt–alkali stress, plants mitigate adverse effects through strategies such as the accumulation of organic osmotic regulators, exclusion of excess ions (such as Na^+^ and Cl^−^), acidification of the rhizosphere, and scavenging of reactive oxygen species (ROS).

Saline–alkali stress disrupts ion absorption and transport, leading to nutritional imbalance and metabolic disorders in plants [4]. Under these conditions, plants enhance the activity of P-type and V-type H^+^-ATPase through Na^+^ transport, which pumps H^+^ into the cell and excretes Na^+^, thereby maintaining intracellular ion balance and adapting to the stress [5]. Moreover, the high-pH alkaline environment triggers the secretion of organic acids by plant roots, which combine with alkaline ions to maintain acid–base balance and reduce internal disruptions, a mechanism distinct from salt stress [6,7]. Additionally, saline–alkali stress also triggers oxidative stress, resulting in the accumulation of ROS. Plants reduce ROS accumulation through their antioxidant systems, alleviating oxidative damage and protecting cell membrane structure [8,9,10]. Overexpression of the *GmPKS4* gene can activate the ROS scavenging system and reduce the excessive accumulation of ROS to improve the salt–alkali tolerance of soybeans [11]. Under alkali stress, the cell membrane is damaged, and the malondialdehyde (MDA) and H_2_O_2_ contents in rice are accumulated, thus activating the antioxidant defense system of rice. External application of natural anthocyanin solution could alleviate the damage of rice leaf wilting and chlorophyll content reduction and effectively remove the excessive accumulation of ROS [12]. Under high salt stress, the antioxidant enzyme activity of sorghum seedlings will be significantly enhanced with the increase in alkaline concentration [13]. Increasing the activity of antioxidant enzymes and the content of antioxidants can prevent the excessive accumulation of ROS and reduce the oxidative damage caused by saline–alkali stress.

NARK kinase regulates plant signal transduction, nodule formation, and abiotic stress responses. Its kinase domain controls cell growth through phosphorylation and dephosphorylation [14]. Specifically, GmNARK plays a critical role in the self-regulation of nitrogen fixation in soybeans by employing a negative feedback mechanism to limit nodule formation and maintain the balance of the nitrogen fixation system [15]. GmNARK suppresses key nodulation transcription factors such as GmNIN, inhibits nodulation signaling, promotes lateral root development to balance root and nodule growth, and enhances plant immune defenses against rhizobium infection [16]. In addition, GmNARK kinase not only plays an important role in symbiotic nitrogen fixation in soybeans but also participates in regulating multiple physiological processes such as soybean root development, plant growth, hormone regulation, and stress responses [17]. *GmNARK* has completely different functions in the plant response to salt and ABA stresses, and overexpressing *GmNARK* in *Arabidopsis thaliana* increases the plant’s tolerance to ABA and salt stresses, indicating that this gene is a candidate gene for breeding stress-tolerant varieties and exerts its important functions in regulating plant growth and development [17]. Studies have shown that abiotic stress can lead to the deformation of soybean root hair and reduce the number of soybean nodules [18]. In terms of hormone regulation function, signaling in GmNARK-mediated nodule inhibition plays an inhibitory role in leaf jasmonic acid (JA) biosynthesis, which is an important step in self-signaling regulation [19]. Because soybean has high genetic diversity, such as strong stress resistance, salt–alkali resistance, disease resistance, and drought tolerance, it is still necessary to continue to study various functions and mechanisms of the *GmNARK* gene in soybean, which is of great significance to improve crop varieties and improve soybean germplasm resources.

In this study, we aim to investigate the role of *GmNARK* in enhancing soybeans’ tolerance to alkali stress, focusing on its involvement in maintaining ROS homeostasis and regulating organic acid secretion in roots to stabilize the internal pH. We found that GmNARK, by modulating antioxidant enzyme activities and influencing the secretion of organic acids in response to alkaline stress, helps mitigate oxidative damage and maintain cellular functions, thereby improving plant growth and stress tolerance. This research seeks to provide new insights into the molecular mechanisms underlying abiotic stress responses in soybeans, with potential implications for improving crop resilience under challenging environmental conditions.

## 2. Results

### 2.1. Expression of GmNARK Gene Induced by Alkali Stress

**Figure 1:**
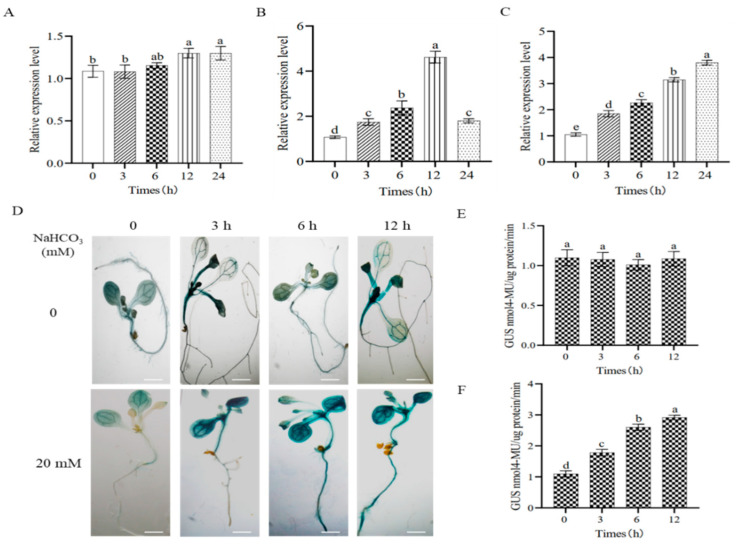
Expression patterns and responses of *GmNARK* to alkaline stress. (**A**–**C**) The spatial expression patterns of the *GmNARK* gene under NaHCO_3_ stress determined by RT-qPCR analyses. *GmGAPDH* was selected as an internal reference gene. The values of mean ± standard error and *t-*test were performed for multiple comparisons with three independent biological replicates using one-way ANOVA. (**D**–**F**) The qualitative and quantitative analyses of GUS activities were performed according to the protocols. Bars = 100 μm. Each figure shown here represents a representative result from three independent experiments. Error bars represent the mean ± SE of three biological replicates, and different letters indicate significant differences by one-way ANOVA (*p* < 0.05).

The previous studies have shown that the protein sequence homology between GmNARK and AtCLV1 is 77.24%, and the promoter of *GmNARK* contains a stress response element (ATTCTCTAAC) that may respond to abiotic stress [17]. Different from salt stress, alkali stress mainly inhibits plant growth through a high pH environment. In this study, we investigated the expression level of *GmNARK* under 100 mM NaHCO_3_ conditions. The RT-qPCR data showed that the expression levels of the GmNARK gene in root and leaf tissues were significantly increased (Figure 1). In root tissue, the transcription level of the *GmNARK* gene was upregulated by alkaline stress and reached the highest level at 12 h (Figure 1B). In leaves, the transcription level of the *GmNARK* gene was also upregulated by alkaline stress, with the highest expression at 24 h (Figure 1C). We then performed GUS staining analysis using transgenic Arabidopsis lines carrying Pro*_GmNARK_::GUS* (Figure 1D). Under normal conditions, the leaves and roots of the transgenic seedlings are light blue, and their GUS activities did not show significant differences. However, after alkali stress treatment, the seedlings changed to a darker blue with stress time duration, and the roots showed the darkest color. At 12 h of alkali treatment, GUS activity reached the highest (Figure 1F). Consistent with the histochemical and biochemical data, the RT-qPCR analyses confirm that the *GmNARK* gene responded to alkaline stress.

### 2.2. Overexpression of GmNARK Improves Alkali Tolerance in Transgenic Arabidopsis

**Figure 2:**
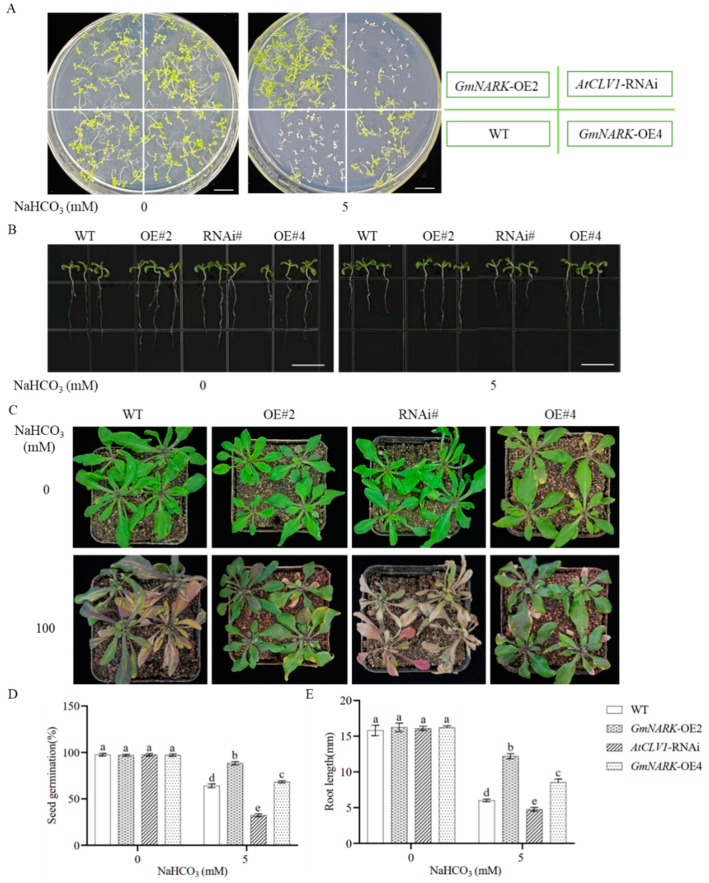
Function of *GmNARK* gene in regulating Arabidopsis resistance to alkaline. (**A**) Comparison of seed germination rates of WT, *GmNARK* overexpression, and *AtCLV1*-RNAi Arabidopsis lines under alkaline stress. Bars = 10 mm. (**B**) Comparison of growth phenotypes of Arabidopsis lines under alkali stress. Bars = 15 mm. (**C**) Phenotypes of Arabidopsis lines after alkali treatment. (**D**) Statistical data of seed germination rates under alkali stress. (**E**) Statistical data of root length. Each figure shown here represents a representative result from three independent experiments. Error bars represent the mean ± SE of three biological replicates, and different letters indicate significant differences by one-way ANOVA (*p* < 0.05).

The above data indicated that *GmNARK* could be induced by alkali stress. We predict that *GmNARK* may play a certain role in plant resistance to alkaline stress. To aim at this goal, we obtained *GmNARK* overexpression Arabidopsis lines. At the same time, *AtCLV1*, is a homologous gene of the soybean *GmNARK* gene in Arabidopsis. The expression levels of *Gm-NARK* and *AtCLV1* genes in Arabidopsis overexpression and knockdown lines were verified by RT-qPCR analyses (Figure S1A,B). First of all, we compared the germination rates of different Arabidopsis lines on 1/2 MS media with or without 5 mM NaHCO_3_. The seed germination data showed that all the Arabidopsis lines had equal germination rates on the control medium but demonstrated significant seed germination rates on the medium with alkaline stress. The WT, *GmNARK* overexpression, and *AtCLV1-*RNAi seeds had about 60%, 90%, and 30% germination rates, respectively. The cotyledons of *AtCLV1-*RNAi seedlings showed significant chlorosis with delayed root emergence (Figure 2A,D). The germinated seeds were transferred onto 1/2 MS media with or without alkaline stress for further growth. After growing for a few days, the plant phenotypes were investigated. The data showed that overexpression of the *GmNARK* gene and silencing of the *AtCLV1* gene significantly promoted or inhibited plant growth compared to the WT line (Figure 2B,E). At the same time, the seedlings were transferred into potting soils for 3 weeks. The plants were irrigated with water or 100 mM NaHCO_3_ solution for a time. After a week, the plant growths were observed. The WT plants, especially *AtCLV1-*RNAi plants, showed severe wilting and chlorosis. The *GmNARK* overexpression plants exhibited healthy, bearing growth with green leaves with anthocyanin accumulation to some extent (Figure 2C), indicating that the soybean *GmNARK*, as well as its homolog *AtCLV1*, could positively regulate Arabidopsis tolerance to alkaline stress.

### 2.3. Overexpression of GmNARK Enhanced Alkali Tolerance of Transgenic Soybeans

**Figure 3:**
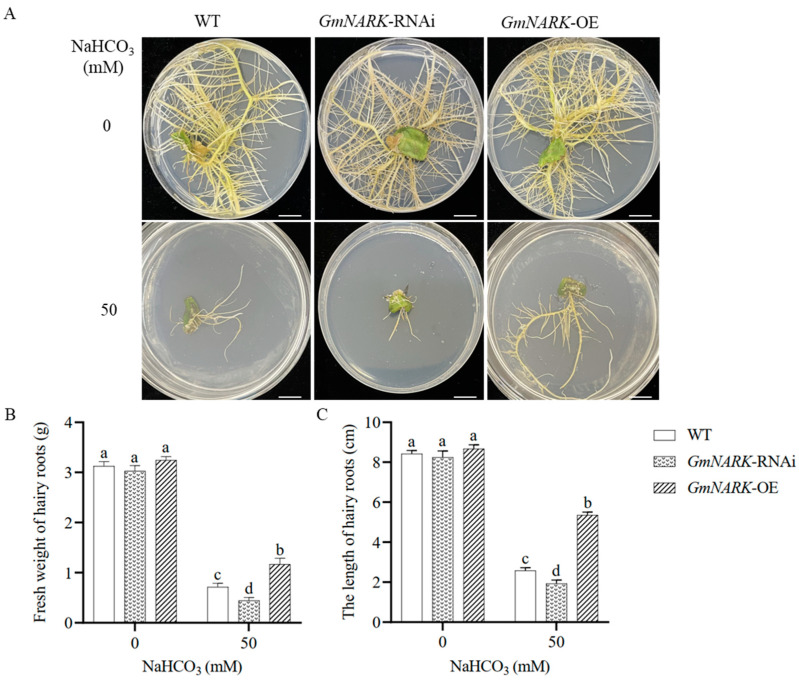
Overexpression of *GmNARK* in hairy roots improved alkali tolerance of transgenic composite soybeans. (**A**) Growth of different transgenic soybean hairy roots under normal and stress conditions. Bars = 15 mm. (**B**) Comparison of fresh weights of hairy roots of different transgenic soybean lines under normal and alkaline conditions. (**C**) Comparison of hairy root lengths of different transgenic soybean lines under normal and alkaline conditions. Each figure shown here represents a representative result from three independent experiments. Error bars represent the mean ± SE of three biological replicates, and different letters indicate significant differences by one-way ANOVA (*p* < 0.05).

In order to further verify *GmNARK* function, we overexpressed the *GmNARK-GFP* gene in the induced hairy roots by agrobacterium-mediated cotyledon-node infection. The gene expression level was detected by the RT-qPCR method (Figure S2A), and the GFP fluorescence in the root tips was observed under fluorescent light (Figure S2C). The *AtCLV1*-RNAi plants were selected for further analysis (Figure S2B). In order to evaluate the *GmNARK* function in soybean resistance to alkali stress, we grew the transgenic composite soybean plants carrying empty vector, *GmNARK-GFP*, and *GmNARK*-RNAi constructs on the plates with or without 50 mM NaHCO_3_. After 2 weeks of incubation, the plant growths were observed and analyzed. The data showed that the different lines exhibited similarly healthy growth on the control plates (0 mM NaHCO_3_), whereas the transgenic lines of Pro*_35S_::GmNARK-GFP* had the longest roots, and the transgenic lines of *GmNARK*-RNAi had the shortest roots (Figure 3A–C), suggesting that GmNARK plays a positive role in regulating soybean resistance to alkaline stress, which is consistent with its function in transgenic Arabidopsis.

### 2.4. Overexpression of GmNARK Enhanced the Secretion of Organic Acids

**Figure 4:**
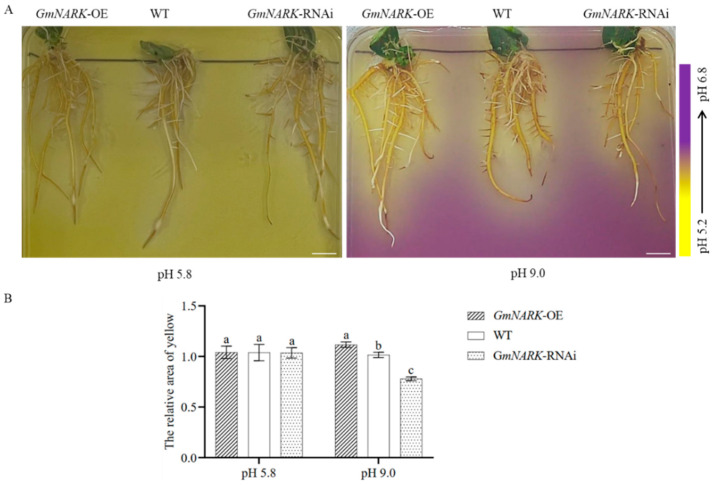
Analyses of rhizosphere acidification in hairy roots of different soybean lines. (**A**) Determination of rhizosphere acidification in soybean hairy roots. Bars = 5 mm. (**B**) Statistical analyses of rhizosphere acidification by measuring and comparing sizes and intensities of yellow areas in rhizospheres. Each figure shown here represents a representative result from three independent experiments. Error bars represent the mean ± SE of three biological replicates, and different letters indicate significant differences by one-way ANOVA (*p* < 0.05).

When plants are subjected to alkali stress, as a response, the rhizosphere of plants often secretes acidic substances to neutralize the alkaline environment so that plants are less damaged by high pH and maintain normal plant growth and development. Therefore, we simulated alkali stress and further determined the acidic substances secreted by the hairy roots of different transgenic soybean lines (Figure 4A). Under alkaline conditions (pH 9.0), the acidified yellow area around the rhizosphere of *GmNARK* overexpression hairy roots was relatively larger than those of the WT and *GmNARK*-RNAi lines. After quantitative measurement and statistical analysis (Figure 4B), we can see that GmNARK can play a significant role in promoting root secretion of acidic substances to neutralize alkaline stress in the rhizosphere environment.

**Figure 5:**
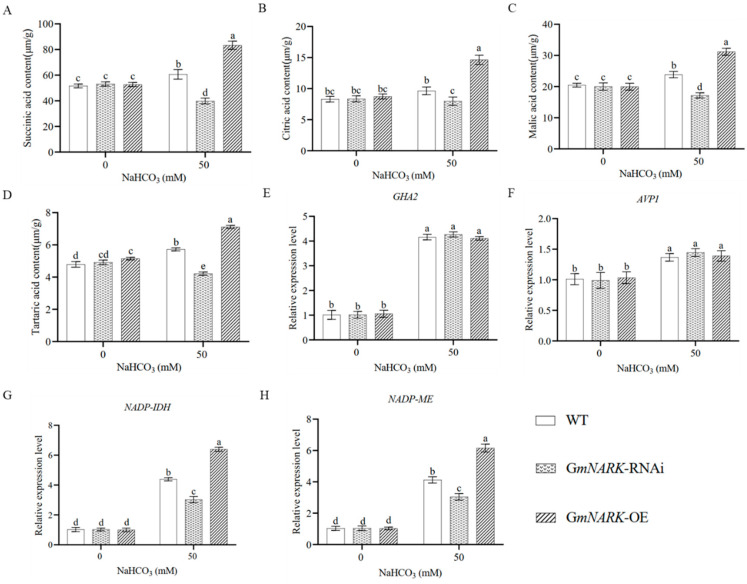
Promotion of organic acid contents and the related gene expression levels by GmNARK kinase in response to alkali stress. Contents of organic acids (succinic acid, citric acid, malic acid, and tartaric acid) in hairy roots of transgenic *GmNARK* soybeans under normal and alkaline conditions (**A**–**D**). Transcriptional levels of alkaline stress-related genes (*GHA2*, *AVP1*, *NADP-IDH*, and *NADP-ME*) in hairy roots of transgenic *GmNARK* soybeans under normal and alkaline conditions (**E**–**H**). Each figure shown here represents a representative result from three independent experiments. Error bars represent the mean ± SE of three biological replicates, and different letters indicate significant differences by one-way ANOVA (*p* < 0.05).

From the above results, we can see that GmNARK kinase can promote roots to secrete organic acids to maintain the acid–base balance in the rhizosphere when soybean plants are subjected to alkali stress. Therefore, we then determined the contents of succinic acid, citric acid, malic acid, and tartaric acid in the hairy roots of different soybean lines by HPLC (Figure 5A–D). The data showed that the contents of these four organic acids in hairy roots with overexpression of *GmNARK* were significantly higher than those of WT, and the contents of organic acids in hairy roots with *GmNARK*-RNAi were significantly lower than those of WT under alkali stress. These results indicate that the hairy roots overexpressing *GmNARK* adapt to alkali stress by secreting organic acids and neutralizing high pH environments. To further investigate how GmNARK increases the tolerance of transgenic soybean hairy roots to an alkaline stress environment simulated by NaHCO_3_, we measured the expression patterns of four alkaline stress-responsive genes: *GHA2* (H^+^-ATPase gene), *AVP1* (inorganic pyrophosphatase gene), *NADP-IDH* (isocitrate dehydrogenase gene), and *NADP-ME* (malaise gene) (Figure 5E–H). The RT-qPCR data showed that the transcription levels of *NADP-IDH* and *NADP-ME* genes were upregulated in *GmNARK* overexpression plants and downregulated in *GmNARK*-RNAi plants, and there was no significant difference in the expression levels of *GHA2* and *AVP1* genes between normal and alkali stress conditions. These data further indicate that the major role of GmNARK kinase in the enhancement of soybean resistance to alkaline stress is to promote roots to secrete organic acids and neutralize alkali stress in the rhizosphere.

### 2.5. Overexpression of GmNARK Improved Soybean Tolerance and Inhibited Soybean Nodulation Under Alkali Stress

**Figure 6:**
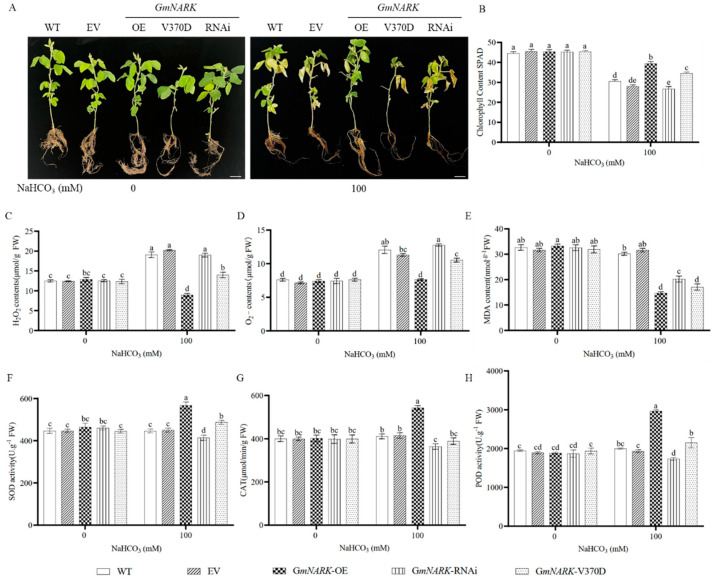
Phenotype and physiological indexes of transgenic soybean lines. (**A**) Effect of alkali stress on growth of different soybean lines. Bars = 10 mm. (**B**) Statistical data of chlorophyll contents in soybean leaves. (**C**) H_2_O_2_ contents in the hairy roots of different soybean lines. (**D**) Superoxide anion (O_2_⁻) contents in the hairy roots of different soybean lines. (**E**) MDA contents in the hairy roots of different soybean lines. (**F**–**H**) Enzymatic activities of SOD, CAT, and POD in the hairy roots of different soybean lines. Each figure shown here represents a representative result from three independent experiments. Error bars represent the mean ± SE of three biological replicates, and different letters indicate significant differences by one-way ANOVA (*p* < 0.05).

To investigate whether GmNARK kinase promotes tolerance of adult soybean plants to alkaline stress or not, we treated the transgenic plants with water or 100 mM NaHCO_3_ solution for one week (Figure 6A). The results showed that all soybean lines exhibited similar growth under normal conditions but demonstrated very different growth under alkaline conditions. The *GmNARK*-RNAi plants showed weak growth with yellowish leaves. The plants expressing the super-nodulation mutant *GmNARK*(V370D) (with an amino acid mutation at position 370) showed the weakest growth with wilted leaves. In contrast, *GmNARK* overexpression soybean plants showed the strongest growth with the most greenish and healthy leaves, demonstrating enhanced alkali tolerance. When plants are subjected to alkali stress, the stress can cause oxidative stress reactions and subsequently produce a large amount of ROS, which will damage the plasma membrane and DNA of plant cells [20,21]. At the same time, as signal molecules, ROS can stimulate downstream signals to stimulate programmed cell death and apoptosis. Then, we measured the contents of H_2_O_2_, O_2_˙^−^, and MDA in the different soybean lines. As shown in Figure 6C–E, the contents of ROS molecules were similar among all the soybean lines under normal conditions, whereas the contents varied greatly under alkali conditions. The contents of the three ROS molecules were the lowest in *GmNARK* overexpression soybean plants but were the highest in *GmNARK*(V370D) overexpression, WT, and EV lines. In order to determine whether overexpression of *GmNARK* can stimulate antioxidant metabolism in transgenic composite soybean plants under alkali stress, we measured the activities of antioxidant enzymes such as SOD, POD, and CAT in the roots of all soybean lines. The activities of antioxidant enzymes in *GmNARK* overexpression plants were significantly higher than those of all other soybean lines after the plants were subjected to alkaline stress (Figure 6F–H).

In order to further explore the mechanism of how GmNARK kinase positively regulates soybean response to alkali stress, we used the RT-qPCR method to investigate the expression patterns of stress-related genes in the transgenic hairy roots.

**Figure 7:**
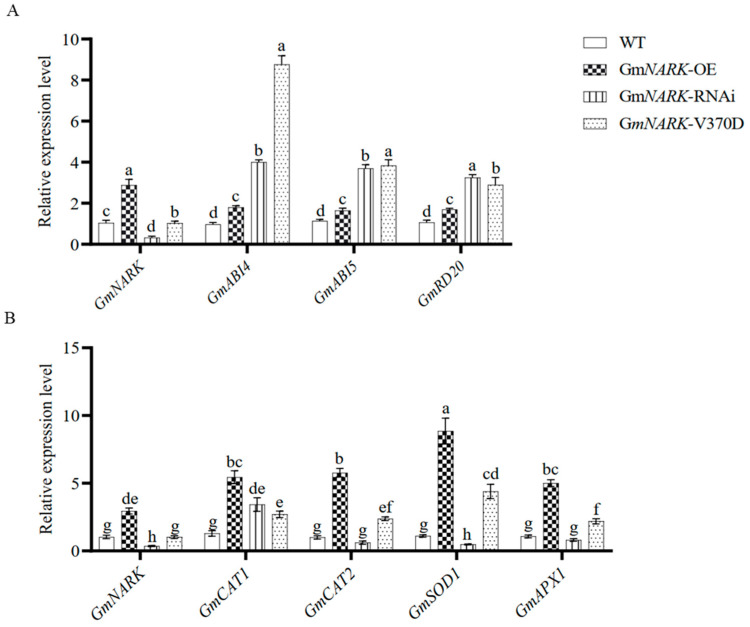
Analysis of related gene expression in soybean hairy roots treated with NaHCO_3_. (**A**) Transcription levels of ABA signaling pathway genes in soybean hairy roots. (**B**) Transcription levels of genes related to the ROS signaling pathway in soybean hairy roots. Each figure shown here represents a representative result from three independent experiments. Error bars represent the mean ± SE of three biological replicates, and different letters indicate significant differences by one-way ANOVA (*p* < 0.05).

As shown in Figure 7, although significant differences were observed in the expression levels of ABA signal transduction-related genes, such as *GmABI4*, *GmABI5*, and *GmRD20*, in soybean plants with *GmNARK* overexpression, *GmNARK*-RNAi, and *GmNARK*(V370D) overexpression after alkali stress treatment (Figure 7A), the RT-qPCR results suggest that GmNARK kinase may not be involved in the ABA signaling pathway. In contrast, the expression levels of antioxidant enzyme genes, such as the Cu/Zn superoxide dismutase gene *GmSOD1* [22], ascorbate peroxidase gene *GmAPX1*, and catalase gene *GmCAT2*, were the highest in GmNARK overexpression plants. In *GmNARK*-RNAi and *GmNARK*(V370D) overexpression plants, the expression levels of these genes were the lowest (Figure 7B). These findings indicate that GmNARK plays a critical role in alkali stress tolerance, likely by enhancing the expression of antioxidant enzyme genes (*GmSOD1*, *GmCAT2*, *GmAPX1*) in the ROS signaling pathways, thereby improving the plants’ ability to tolerate alkali stress.

## 3. Discussion

The soybean GmNARK gene encodes a CLAVATA 1 (CLV1)-like receptor kinase, which is a key regulator of AON (nodulation autoregulation) [23]. The mutant of *GmNARK* presents a super-nodulation phenotype and plays a key role in controlling the number of soybean nodules. Although its functions in regulating plant nodulation have been extensively elucidated, as a receptor-like kinase enriched with leucine-rich repeats, GmNARK’s roles in regulating plant growth, development, and responses to abiotic stress hormones remain elusive [16]. Previous studies have indicated that UV-B and stresses can regulate nodule formation in leguminous plants, with varying UV-B intensities enhancing soybean nodule formation [24]. Salt stress-induced reactive oxygen species (ROS) can trigger plasma membrane endocytosis and lead to localization changes, subsequently initiating a series of physiological and biochemical responses [25]. GmNARK is located on the plasma membranes, and its function may be altered by endocytosis-mediated vesicle trafficking induced by NaCl treatment [17]. In Arabidopsis, overexpression of *GmNARK* increased sensitivity to ABA and NaCl during seed germination and post-germination stages. Moreover, *GmNARK* positively regulated the expression of ABA-responsive genes in these transgenic plants [17]. In this study, we found that the soybean GmNARK kinase responds to alkali stress. Overexpression of *GmNARK* significantly enhanced alkali tolerance in both transgenic Arabidopsis and composite soybean plants, suggesting that NARK kinase may regulate plant responses to salt and alkaline stresses through different mechanisms. The *GmNARK* gene is expressed in the root, stem, and leaf tissues of soybeans. Nontachaiyapoom et al. reported the specific expression of *GmNARK* in sieve tube cells through promoter analysis and GUS reporter gene assays [26]. The root is the primary organ that directly contacts the external soil environments, and it serves as the main location for the exchange of water and ions [27]. Under alkaline stress, the roots can secrete acidic substances to maintain the pH balance with the external alkaline environment [28]. As a key channel for internal material transport and signal transduction, *GmNARK* expression in phloem helps coordinate root cell activity, precisely regulates the secretion of acidic substances, and maintains overall plant pH stability, thereby enhancing alkaline stress tolerance [29]. In this study, we measured organic acid contents and analyzed transcription levels of the related genes, such as H⁺-ATPase. Our results showed that overexpression of *GmNARK* significantly increased the accumulation of organic acids in soybean roots, positively regulating the plant’s alkaline tolerance. When *GmNARK* overexpression soybeans were subjected to alkaline stress, the levels of succinic acid, malic acid, citric acid, and tartaric acid in both the leaves and roots were higher than those in the other lines. Notably, the genes for organic acids were also higher in the *GmNARK* overexpression transgenic lines than those in the other lines (Figure 5). The synthesis and accumulation of organic acids in plants is an important mechanism for responding to alkaline stress and regulating the pH balance between plants and their living environments [30]. Further analyses revealed that the transcription levels of *NADP-IDH* and *NADP-ME* genes were significantly upregulated in *GmNARK* overexpression transgenic lines, confirming that GmNARK participates in helping plants adapt to alkaline stress. The chlorophyll content is a key indicator of plants’ physiological status, directly reflecting the plant’s photosynthetic capacity [31]. Environmental stresses, such as drought, salinity, disease, and pests, negatively affect chlorophyll contents [32,33]. Our study showed that the *GmNARK* overexpression transgenic lines possessed the highest chlorophyll contents among all the soybean lines under alkaline stress (Figure 6B).

Abiotic stresses, such as alkalinity, can lead to the accumulation of reactive oxygen species (ROS) in plant cells. ROS are highly reactive oxygen derivatives, including superoxide anions (O_2_˙^−^), hydroxyl radicals (OH), hydroperoxyl radicals (HO_2_), and hydrogen peroxide (H_2_O_2_) [34]. In plant cells, ROS function as a double-edged sword. At low concentrations, they act as signaling molecules, promoting growth and development. However, under stress conditions, the balance between ROS production and scavenging is disrupted, leading to excessive ROS accumulation. This imbalance can damage cell membranes and other cellular components, negatively affecting plant growth [35]. ROS accumulation not only triggers stress responses at the whole-plant level but also exerts different effects in different organs and cells. The regulation of ROS in plant cells depends on specific organelles and their functions, such as mitochondria, chloroplasts, and peroxisomes [36]. For example, in chloroplasts, O_2_˙^−^ and H_2_O_2_ produced by photosystem I can be dismutated by superoxide dismutases (SODs) in the stroma of the thylakoid membrane or spontaneously form H_2_O_2_. H_2_O_2_ can be detoxified by ascorbate peroxidases (APXs), glutathione peroxidases (GPXs), and peroxiredoxins (PrxRs) [37]. Therefore, plants maintain ROS homeostasis by regulating the antioxidant enzyme system, thereby coping with stress conditions. For instance, overexpression of the *SENESCENCE-SUPPRESSED PROTEIN PHOSPHATASE* (*SSPP*) gene in Arabidopsis and soybean reduced ROS levels and enhanced salt stress tolerance [38]. Similarly, the transgenic soybean plants overexpressing *GsWRKY40*-*GsbHLH92* genes exhibited reduced ROS accumulation and improved salt tolerance [39]. In leguminous plants, ROS also play a key role in regulating nodulation. Reduced ROS production leads to downregulation of nodulation-related genes [40,41,42]. As a nodulation receptor kinase, GmNARK helps maintain ROS homeostasis and alleviates oxidative stress caused by alkaline conditions. In our study, no significant differences in H_2_O_2_ and MDA levels were observed between *GmNARK* overexpression and WT plants in normal conditions. However, under NaHCO₃ conditions, the *GmNARK* overexpression plants accumulated higher levels of H_2_O_2_, O_2_˙^−^, and MDA, indicating an enhanced response to oxidative stress. Moreover, SOD, CAT, and POD activities were significantly increased in *GmNARK* overexpression lines compared to WT. Similar findings have been reported in rice, where the key antioxidant genes, such as *OsCu/Zn-SOD*, *OsAPX2,* and *OsCAT1*, were upregulated in *OsSAP6* overexpression lines under alkaline stress [43]. In maize, overexpression of transcription factor *ZmNF-YA8* improved salt tolerance by activating the antioxidant enzyme gene *PEROXIDASE 1* (*ZmPER1*) to reduce ROS levels [44]. We hypothesize that the enhanced antioxidant activities observed in *GmNARK* overexpression plants are due to the upregulation of antioxidant enzyme genes, such as *GmSOD1*, *GmCAT2*, and *GmAPX1*, which can bolster the plant’s ROS-scavenging capacities. This reduction in oxidative damage to key cellular components, including proteins, membrane lipids, and DNA [45], helps maintain cellular integrity, improve growth, and ultimately increase tolerance to alkaline stress.

In conclusion, GmNARK plays a multifaceted role in regulating plant responses to alkaline stress. It enhances the synthesis of organic acids in the roots to maintain internal and external pH balances and also preserves ROS homeostasis by modulating the expression of antioxidant enzyme genes. These two mechanisms work together, forming a complex regulatory network. GmNARK thus ensures plant survival under alkaline stress by reducing damage and sustaining normal growth through coordinated physiological and biochemical processes.

## 4. Materials and Methods

### 4.1. Plant Material and Stress Treatment

*Arabidopsis thaliana* ecotype (Col-0) and cultivated soybean Williams 82, both stored in the Key Laboratory of Agricultural Biological Functional Genes, Northeast Agricultural University, were used in this study. After surface sterilization, *Arabidopsis* seeds were inoculated on 1/2 MS medium with or without 5 mM NaHCO_3_, vernalized in the dark at 4 °C for 2 days, and then grown in a plant growth chamber (22 °C, 60% relative humidity, 16 h of light, 8 h of dark cycle). Seed germination rate, seedling root length, and phenotype observation at the seedling stage were recorded. Soybean Williams 82 seeds were sown in pots containing vermiculite and grown in a soybean climate chamber (26 °C, 50% relative humidity, 16 h of light, 8 h of darkness cycle). After 6 days, the seedlings of Williams 82 were treated with 100 mM NaHCO_3_ simulating the alkali stress environment. After 0, 3, 6, 12, and 24 h, the leaf and root tissues were collected for RNA extraction, respectively.

### 4.2. Analysis of Spatiotemporal Expression Patterns of the Related Genes

The total RNA samples extracted above were synthesized into cDNA, and the real-time fluorescence quantitative RT-qPCR amplification was carried out by the SYBR dye method to detect the gene expression levels in different soybean tissues. *GAPDH* and *Actin2* served as reference genes for soybean and *Arabidopsis*, respectively. The data were analyzed using the 2^−ΔΔCT^ method.

Pro*GmNARK*::*GUS* transgenic seedlings grown on 1/2 MS medium for 5 days were treated with alkali for different time durations and then were stained for GUS assay. The control and NaHCO_3_-treated Arabidopsis seedlings were stained in GUS staining solution at 37 °C overnight, followed by decolorization with 70% ethanol until the background was sufficiently clear. The seedlings were then rinsed with ddH_2_O to remove any remaining ethanol, transferred to fresh ddH_2_O, and observed under a microscope. Images were taken for documentation.

### 4.3. Construction of RNAi Interference Vector

The backbone vector is pFGC5941. The homologous sequence of the *GmNARK* gene is aligned by NCBI database (accessed on 1 March 2023) and the Phytozome website (accessed on 1 March 2022), and a specific fragment (300 bp) was selected for the generation of the RNAi construct. The obtained plasmid was used for silencing the *GmNARK* gene after sequencing.

### 4.4. Plant Genetic Transformation

The obtained vectors, pBWA(V)BS-*3HA-GmNARK-GFP* and RNAi interfering vector were transformed into *Agrobacterium tumefaciens* strain GV3101. Arabidopsis was transformed using the flower-dipping method, and T_0_ generation seeds were harvested and sown on 1/2 MS medium containing 50 mg/L kanamycin for selection. The surviving seedlings were transplanted into soil, and the T_1_ and T_2_ generation seedlings were sprayed with Basta herbicide to screen for transgenic-positive plants. The expression level of *GmNARK* in transgenic *Arabidopsis* was detected by RT-qPCR assay. The induction and genetic transformation of soybean hairy roots were performed by Agrobacterium-mediated cotyledon node infection.

### 4.5. Analyses of Alkali Tolerance of GmNARK Transgenic Plants

In order to characterize the tolerance of plants to alkali stress, the transgenic *Arabidopsis thaliana* positive plants and WT plants were grown under normal and alkali conditions for 21 days, and their phenotypes were observed and recorded, and the phenotypes of transgenic *Arabidopsis thaliana* before and after treatment were photographed and recorded.

The obtained WT, *GmNARK* overexpression, and *GmNARK-*RNAi transgenic composite soybean lines were transferred to normal rooting agar plates (pH 5.8) and alkaline plates (pH 9.0), respectively, and these agar plates were supplemented with 0.004% bromocresol violet as a pH indicator. The pH change around the roots can be visualized and photographed. When the pH of the medium decreases from neutral to acidic, the color changes from purple to bright yellow, and the color bar shows the chromatogram as a function of pH in the same medium. The yellow-colored areas and intensities were quantified by using ImageJ software (version 1.53).

### 4.6. Determination of Organic Acid Contents

High-performance liquid chromatography (HPLC) was used to determine the organic acid contents of the different transgenic soybean lines. For details, please refer to the HPLC method published by Zhang Pei for the determination of organic acid contents in pulp water [46].

### 4.7. Analyses of ROS and Antioxidant Contents

The content of malondialdehyde (MDA), hydrogen peroxide (H_2_O_2_), and superoxide anion (O_2_˙^−^), as well as the activities of catalase (CAT), peroxidase (POD), and superoxide dismutase (SOD), were measured using the corresponding assay kits (Boxbio, Beijing, China) according to the manufacturer’s instructions [31,47].

### 4.8. Analysis of Expression Levels of Genes Involved in ABA and ROS Pathways

Gene expression patterns of the stress-related genes were investigated in transgenic hairy roots using a RT-qPCR assay. The expression levels of ABA signaling pathway genes (*GmABI4*, *GmABI5*, *GmRD20*) [48,49,50] and ROS pathway genes (*GmSOD1*, *GmCAT1*, *GmCAT2, GmAPX1*) [22,38] were measured under 100 mM NaHCO_3_ treatment. The data were analyzed using the 2^−ΔΔCT^ method.

### 4.9. Statistical Analyses

All experiments in this study were repeated at least three times, and the mean of the three measurements was used for analysis. The relative quantitative analysis of gene expression was carried out by the 2^−ΔΔCT^ relative quantitative method. Using Data Processing System (DPS) statistical software, version 15 [51]. GraphPad Prism version 7 for Windows, GraphPad Software, Boston, MA, USA, (www.graphpad.com) was used for drawing (accessed on 20 February 2024). Statistical analysis was performed using one-way ANOVA (*p* < 0.05), followed by Tukey’s multiple comparison test to assess pairwise differences between groups. Different letters indicate significant differences among the groups.

## 5. Conclusions

This study revealed the function of GmNARK in regulating plant tolerance to alkali stress, confirmed that soybean nodule receptor kinase GmNARK can regulate plant growth under saline–alkali stress, and clarified that GmNARK can improve plant tolerance to saline–alkali stress by enhancing the generation of organic acids in roots and increasing the expression of genes related to ROS signaling pathways, providing an important theoretical basis for further characterizing the function of GmNARK kinase in the process of stress resistance.

## Data Availability

The data that support the findings of this study are available from the corresponding author upon reasonable request.

## Supplementary Materials

The following supporting information can be downloaded at: https://www.mdpi.com/article/10.3390/ijms26010325/s1.
