# Supplementary material for: Function of Nodulation-Associated GmNARK Kinase in Soybean Alkali Tolerance
Source: Int J Mol Sci. 2025 Jan 2;26(1):325. doi: 10.3390/ijms26010325 (PMC11719578; doi:10.3390/ijms26010325)
Supplement: Supplementary file 1 [file ijms-26-00325-s001.zip › ijms-3311724-supplementary.pdf]

Supplementary materials

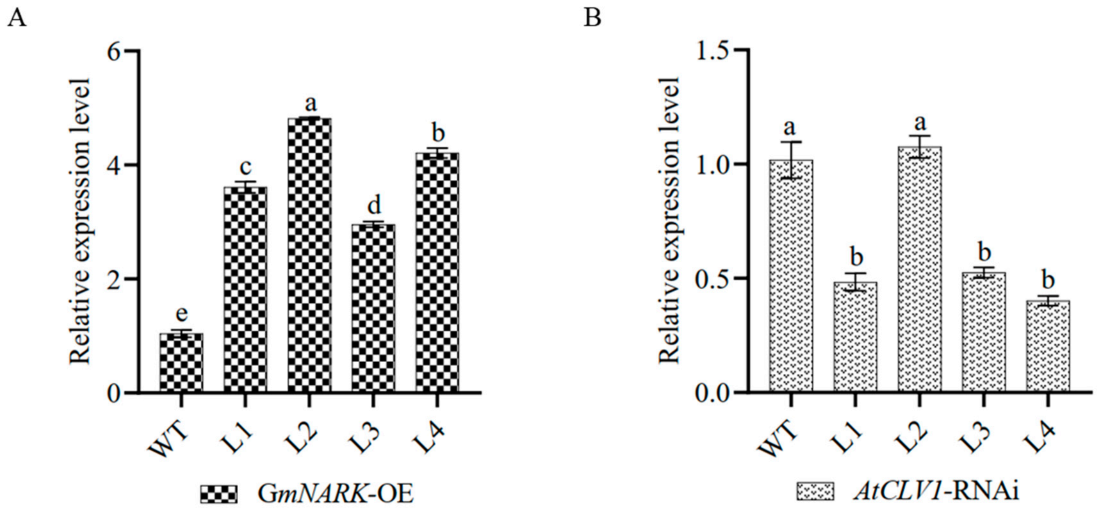

Figure S1 Identification of positive transgenic *Arabidopsis thaliana* (A) Identification of positive plants overexpressing *GmNARK* transgenic *Arabidopsis thaliana* by RT-qPCR assay. (B) Identification of positive plants of *AtCLV1*-RNAi transgenic *Arabidopsis thaliana* by RT-qPCR.

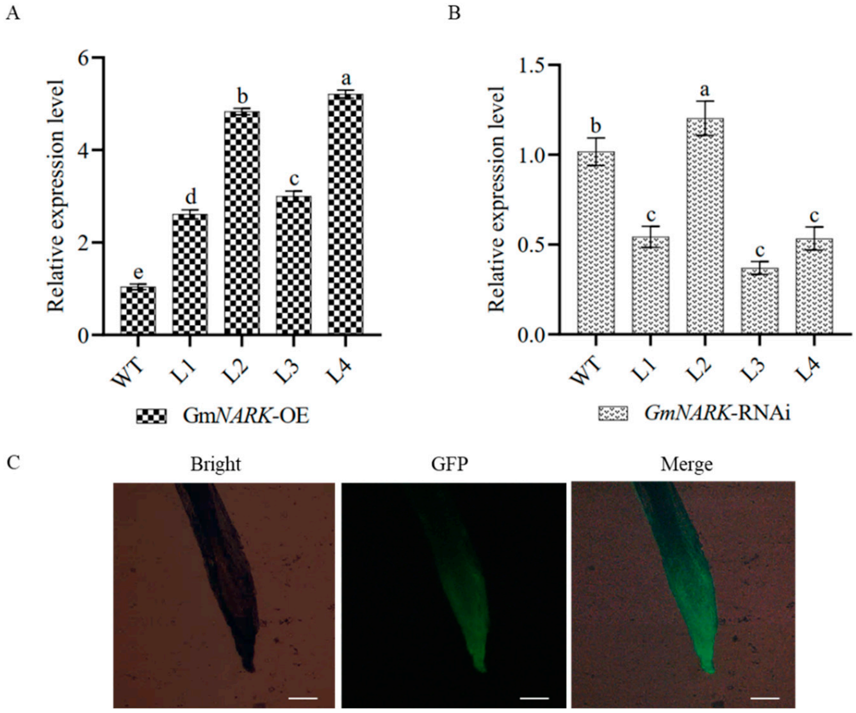

Figure S2 Identification of positive soybean hairy roots (A) Identification of positive plants of transgenic hairy roots overexpressing *GmNARK* by RT-qPCR assay. (B) Identification of positive plants with transgenic hairy roots of *GmNARK*-RNAi by RT-qPCR assay. (C) Identification of positive *GmNARK*-overexpression using GFP green fluorescence images. Bars=2 mm.
